# Updated Egyptian national guidelines for management of hemophilia A in children & adolescents

**DOI:** 10.1007/s00277-025-06557-x

**Published:** 2025-10-09

**Authors:** Galila Mokhtar, Amal El-Beshlawy, Mohsen El Alfy, Magdy El Ekiaby, Magda Rakha, Ahmed Mansour, Azza A.G. Tantawy, Hoda Hassab, Usama El Safy, Khaled A. Eid, Naglaa Shaheen, Naglaa Omar, Sonia Adolf, Seham Ragab, Mohamed ElKholy, Nayera H.K. Elsherif

**Affiliations:** 1 Pediatric Hematology and Oncology unit, Pediatric Department, Ain Shams University, Cairo, Egypt; 2 Pediatric Hematology Unit, Pediatric Department, Cairo University, Giza, Egypt; 3 Shabrawishi Hospital International, Hemophilia Treatment Center, Giza, Egypt; 4 Senior Consultant Hematologist, President of the Egyptian Society of Hemophilia, Cairo, Egypt; 5 Pediatric Hematology and Oncology unit, Pediatric Department, Mansoura University, Monsoura, Egypt; 6 Pediatric Hematology and Oncology unit, Pediatric Department, Alexandria University, Alexandria, Egypt; 7 Pediatric Hematology and Oncology unit, Pediatric Department, Zagazig University, Zagazig, Egypt; 8 Pediatric Hematology, Cairo University, Cairo, Egypt; 9 National Health Insurance Organization, Cairo, Egypt; 10 Pediatric Hematology Consultant, Cairo University, Cairo, Egypt; 11 Pediatric Hematology, Pediatric Department National Research Center, Cairo, Egypt; 12 Pediatric Hematology and Oncology unit, Pediatric Department, Menoufia University, Menoufia, Egypt; 13 Clinical Hematology/Internal Medicine Department, Cairo University, Cairo, Egypt

**Keywords:** Hemophilia A; central nervous system bleeding, Obesity, Guidelines, Circumcision, Transitional care, Hemophilia carrier

## Abstract

To improve patient advocacy in hemophilia, the World Federation of Hemophilia (WFH) recommends establishing a National Hemophilia Committee (NHC), developing standards and guidelines, broadening the community through including people with von Willebrand disease, other rare bleeding disorders and carriers of hemophilia. In 2018, the Egyptian Society of Hemophilia (ESH) took the initiative, developed, and published guidelines for Hemophilia A care [1] (Table 1). Aim & Methods given the cmanagement, the panel `s goal was to update the ESH guidelines for Hemophilia A care using the modified Delphi method to address unmet needs and local requirements in Egyptian healthcare settings and include management of obesity in hemophilia patients, oral health promotion and prophylaxis before dental procedures, routine monitoring of bleeding events and musculoskeletal scoring, treatment of central nervous system bleeding in children and neonates with hemophilia, the management of acquired hemophilia and hemophilia carrier, the use and monitoring of Non-factor replacement in prophylaxis, finally the implementation of transitional care and patient advocacy.

## Introduction

According to 2020 WFH Annual Global Survey the number of registered patients with hemophilia B is one fifth of registered patients with hemophilia A suggesting a prevalence of one case in 50,000 population. Approximately 1 in 5,000 to 1 in 10,000 males worldwide are born with hemophilia A and 1 in 20,000 to 1 in 40,000 with Hemophilia B [2, 3]. In Egypt, based on the WFH Annual Global Survey, there were an estimated 6,233 people with hemophilia (PWH) in 2020; of these, 4,885 cases were hemophilia A, and 1,143 were hemophilia B [2, 3].

Given the challenges in the developing countries and the need to establish a comprehensive multidisciplinary care setting for Hemophilia A patients [4, 5], the Egyptian Society of Hemophilia (ESH) `s aim was to establish and maintain a national registry of IBD; increasing public awareness about IBD, advocating for PWH rights in accessing appropriate healthcare. Twenty-one hemophilia treatment centres across Egypt are affiliated to ESH; and starting from 2020, IBD are among government priorities and the allocated budget is progressively increasing.

Significant advances in hemophilia treatment include the development of gene therapy, extended half-life factor replacement therapies, and novel non-factor replacement Table 1 therapies to improve treatment efficacy, reduce the frequency of infusions, and enhance the overall quality of life for individuals with hemophilia.

**Table 1:** Summary of recommendations of ESH guidelines 2018 [1]

Nutrition and vaccination • Healthcare for all PWH should include nutritional advice and follow-up – calcium, serum iron, ferritin level and vitamins D level. • Routine hepatitis A vaccination in all haemophilic patients > 1 year of age is mandatory • Hepatitis B screening is essential for PWH > 5 years of age, and it is important to revaccinate those who are negative.
Home treatment • Home treatment should be encouraged; however, it is important that regular clinic visits continue to ensure good management of hemophilia A. • First aid measures and antifibrinolytic agents are encouraged as adjuvant therapy. • Parents and patients should be educated on all the steps underlying home treatment to build confidence, ensure safety and promote it as an option for the management of hemophilia A
Physiotherapy •The hemophilia treater should talk directly with the local physiotherapist to provide education on the special needs of treating PWH, specifically regarding the use of factor replacement therapy, prior to the physiotherapy session • Physiotherapy manoeuvres are best carried out immediately after factor infusion • A short and easy scoring system, such as the Hemophilia Joint Health Score (HJHS), should be adopted to evaluate joint health
**Circumcision** • Circumcision of PWH and/or those with a positive family history of hemophilia Ashould not be considered a minor surgical procedure and must be performed under strict conditions. • Administration of clotting factor concentrate (even if minimal) is usually required before circumcision and should be continued during the week following the procedure.
**Prophylaxis versus on demand** • Prophylaxis is the preferred treatment approach for all PWH to preserve musculoskeletal function. • Low-dose (recombinant third generation Factor VIII 40 IU/kg) prophylaxis for Hemophilia A patients two to three times per week is an effective option, but should be individualised according to age, venous access, bleeding phenotype, activity and availability of clotting factor concentrates • For the treatment of very young Hemophilia A children, one option is to start prophylaxis once per week and escalate the frequency depending on bleeding and venous access
**Clotting factor concentrates** • Recombinant clotting factor VIII concentrates are the recommended treatment of choice for Hemophilia A patients in Egypt. If resources are limited, virally inactivated plasma-derived concentrates can be used • Due to the high prevalence of HCV in Egypt, cryoprecipitate, fresh frozen plasma, and solvent detergent filtered cryoprecipitate should only be used in emergency situations where no alternatives are available
**Surgery** • Due to the increased risk of bleeding during surgery, the surgical team should work with the local hemophilia treatment centre to thoroughly plan procedures prior to surgery • Prior to surgery, the haematologist should provide a written detailed treatment plan including duration and dosage of haemostatic therapies, also covering the rehabilitation phase
**Synovectomy** • Non-surgical synovectomy, guided by ultrasound, is the procedure of choice • Chemical synovectomy should be used when appropriate and available. Rifampicin is highly effective and has few side effects; it can be used in an outpatient setting when preceded and followed by factor infusion, analgesics and bed rest.

## Methodology

The modified Delphi technique was used to develop a consensus among a group of experts on management and care of patients with hemophilia A. The modified Delphi method of group interaction avoids the face-to-face discussions, the vulnerability and the mind change in front of others [6–8].

For selecting experts, a significant contribution to the innovation of inherited bleeding disorders (IBDs) treatment in Egypt; at least 10 years of professional experience in the field of hemophilia & IBDs with international publications, leadership roles in national initiatives regarding hemophilia patients and a willingness to contribute were the used criteria.

The final expert panel comprised 14 individuals from 10 hemophilia care institutes in Egypt (AinShams, Cairo, Mansoura, Alexandria, Zagazig, Benha universities, Shabrawishi Hospital, the National Health Insurance Organization, Menoufia University, and the National research center. Experts had reported an average of 15 years of professional experience in hemophilia & IBDs field, ranging from 20 to 50 years, all had participated in all rounds of the Delphi study, and all were trained in writing recommendations. In January 2022, the experts were asked to list at least 10 issues that are most important for hemophilia patients care this included the main challenges and expected potential. Panellists were asked to give as clear justification of each idea as possible. To identify priority areas. Then a web communication begun in January 2023, and participants were asked to rate the research areas on a scale of importance ranging from 1 to 10, with 1 labelled “very low importance” and 10 labelled “very high importance.” They were also asked to identify the most neglected research areas. The agreed items after rating are shown in Table 2. The initial recommendations were developed by the consultants, as assigned by the ESH leads. Recommendations were based on the literature particularly the WFH new guidelines, as well as on the experience and expertise of the panellists. The panellists were not permitted to discuss the drafted recommendations before the modified Delphi process started to avoid bias.

**Table 2:** The items reaching consensus in each category:

Obesity • Dosing & administration of factor replacement. • Screening tools for identification of overweight/obese people with hemophilia • Lifestyle modifications and physical activity • Caloric intake and Pharmacotherapy &Bariatric surgery • Establishing Behavioral Changes.
Oral/Dental care • Oral Health promotion. • Haemostatic management in dental procedures.
Routine Monitoring • Disability monitoring using FISH, • Musculoskeletal monitoring using HJHS • Pain monitoring. • Annual bleeding rate.
CNS bleeding • CNS bleeding in neonate. • Radiological assessment • Factor and non-factor replacement • Prophylaxis
Hemophilia Carriers Recognition and Care
Acquired Hemophilia Treatment &Diagnosis
Non-factor replacement in treatment and prophylaxis. • Indications • Monitoring
Transitional care • Establish Comprehensive care system • Addressing the Cost of care. • Awareness and education
Patient advocacy

Following the drafting of the recommendations, each set of recommendations went through the modified Delphi consensus rounds that were conducted using a Survey sent via email with all responses remaining anonymous except to the independent facilitator who managed the process.

## The consensus definition 

Three rounds of Delphi surveys were permitted to achieve consensus for the recommendations. The minimum response rate was set at 75% of the panelists. The threshold for achieving consensus was 80% of the respondent’s indicating agreement or strong agreement. Statements achieving consensus in the first or second round were not subjected to subsequent rounds. Drafted recommendations that did not achieve consensus after three Table 3 rounds did not appear in the final guidelines and the ESH leads drafted these recommendations for further research [4, 9].

**Table 3:** Suggested peak plasma factor levels and duration of administration in patients with CNS bleeding (with and without significant resource constraint) [4]

Therapy	Hemophilia A		Hemophilia B	
	Desired level (IU/dl)	Duration (days)	Desired level (IU/dl)	Duration (days)
No significant resource constraint				
Initial	80–100	1–7	60–80	1–7
Maintenance	50	8–21	30	8–21
Significant resource constraint				
Initial	50–80	1–3	50–80	1–3
Maintenance	30–50	4–7	30–50	4–7
	20–40	8–14	20–40	8–14

## Recommendations

### 1-Obesity and clinical impact

Increased prevalence of obesity has been observed in both adult and pediatric hemophilia populations, like trends described for the global population [10]. Wilding et al. demonstrated a link between excess adiposity and a reduced range of motion in weight-bearing joints and a faster loss of joint mobility among PWH [10]. Similar findings have been described by Ullman and colleagues who observed significant reductions in the active range of flexion of the knees and elbows among PWH who were overweight or obese [11]. Analysis of a cohort of Dutch hemophilia patients also reported that being overweight/obese was associated with an increased number of joint bleeds and lower limb function [10]. The association between obesity and the frequency of chronic pain as well as the low bone mineral density has also been described [2, 12]. This highlights the need to assess bone health and to take into account the impact of overweight/obesity on bone mineral density among PWH [2]. PWH A and B who were overweight/obese were less likely to administer home-infusion or self-infusion of factor prophylaxis due to the greater difficulty in obtaining venous access to administer treatment; consequently, unable to obtain all of the benefits of home treatment [11].

**Table:** 

Recommendations (Panellist consensus)
-Dosing according to ‘ideal body weight’ for height is recommended to reduce factor consumption and the cost of prophylaxis while maintaining the safety of patients using the formula developed by McEneny-King etal [12]. -Measurement of waist circumference is recommended to be used as a better alternative screening tool for obesity among PWH to BMI and findings are compared to the cutoff for children and adolescents [13].

### 1.4 Application of general guidelines for weight management in hemophilia

#### 1.4.1 Lifestyle modifications & physical activity

Comprehensive lifestyle modification forms the basis of interventions to prevent weight gain or promote weight loss. More intensive approaches for weight loss, such as pharmacotherapy and bariatric surgery, are recommended for people with obesity-related comorbid conditions and/or higher body weight. Appropriate hemostatic coverage during surgery should be administered in the perioperative setting und er the direction of the HTC [14]. 

The hemophilia care team is responsible for advising PWH on physical activities to participate in, and how to adjust their treatment regimens when undertaking exercise and how to manage potential bleeding events that might result from physical activity. However, for PWH, it is essential to carefully consider the person’s risk of bleeding, level of pain and functional impairment when devising an exercise regimen. Low-impact aerobic activities such as swimming, walking or cardiovascular training using an elliptical machine or stationary bike; reducing the intensity of resistance exercises; incorporating a stretching routine; and recognizing and managing the risk of activity-related pain or bleeding are encouraged [15, 16]. 

**Table:** 

Recommendations (Panellist consensus)
-Increasing physical activity should take account of an individual’s risk of bleeding and level of pain and functional impairment according to the published National hemophilia guidelines for safe sports and exercise -A reduction in caloric intake should be the main component of any weight loss intervention balanced with the need to ensure adequate nutrition -Weight and behavioural self-monitoring using food diaries and web application, goal-setting, education, psychological counselling, stress reduction and mobilization of social support - It is important to involve and consult the multi-dsciplinary hemophilia treatment center (HTC) team when applying general weight management guidelines

## 2-Dental care

In Egypt, children with hemophilia have generally received suboptimal dental care, but their oral health requires thorough assessment [17]. Good oral hygiene for PWH should be encouraged to prevent the need for dental work and oral diseases such as gingivitis, dental caries, and periodontal disease, which may cause serious gum bleeding.

### Dental surgery and invasive procedures

According to The WFH Guidelines, hemostasis management for PWH should be individually planned under the advisement of a hematologist before any dental surgery or other invasive procedure within the oral cavity. Systemic or topical antifibrinolytics (i.e., tranexamic acid or epsilon-aminocaproic acid) are effective as adjunct treatment in the management of dental interventions pre- and postoperatively and have the potential to reduce the need for factor replacement therapy, and antibiotics should only be prescribed if clinically indicated for management of infection. Most dental injections can be delivered safely; intramuscular oral injections require systemic haemostatic measures with the use of alternative low-risk routes of delivery such asintra-ligamentary single-tooth anesthesia or buccal infiltration injections [4].

**Table:** 

Recommendations (Panellist consensus)
• Good oral hygiene should be encouraged in PWH • Hemostasis management (including systemic and/or topical antifibrinolytics) should be individually planned with advice from a hematologist before any dental work is undertaken • Most dental injections can be delivered safely in PWH with the use of alternative low-risk routes of delivery • No need to raise factor levels for restorative procedures • For dental extraction & Dento-alveolar or periodontal surgery, raise factor level to 50–60% and continue factor coverage for 1–2 days (postoperative) with parenteral Tranexamic acid 1 h before surgery then orally for 7–10 days after procedure • For Maxillofacial surgery, raising factor level to 100% with a 7–10 days inpatient postoperative stay are needed • Soft diet for 7 days, careful brushing around wound site for minimum of 3–5 days postoperatively and oral course antibiotic prophylaxis for 7 days are recommended for dental extraction & Dento-alveolar or periodontal surgery, and major surgery Maxillofacial surgery

## Routine monitoring

The WFH Guidelines for the Management of Hemophilia (3rd edition) note that “in the management of hemophilia, outcome assessment refers to the use of specific tools designed to monitor an individual’s disease course and to measure the consequences of the disease and its treatment [4].

### Clinical scoring system and monitoring for bleeding events

According to the WFH Guidelines for the Management of Hemophilia (3rd edition) “the most important indicator of the efficacy of hemostatic therapy is the frequency of bleeding, particularly joint and muscle bleeds [4]. The frequency of bleeding is assessed by estimation of annualized bleeding rate (ABR) which is calculated as the number of reported bleeding events divided by the number of months in the reporting time window (8 weeks to 12 months) and multiplied by 12 [18].Other aspects of bleeding events that should be documented includes the site and duration of each bleed, the target joint or non-target joint bleed whether provoked or spontaneous bleed, the response to factor administration and finally the need for hospitalization and its duration [2, 18]. The FISH (Functional Independence Score for patients with Hemophilia) is an objective, performance-based instrument. It has good face validity, content validity and internal consistency. It correlates well with other self-rated activities such as the Stanford Health Assessment Questionnaire (HAQ), the clinical score and the radiological score [19, 20]. Musculoskeletal status monitoring should include the Hemophilia Joint Health Score (HJHS) in children and young adults [21, 22], the Gilbert score in adults and children with established arthropathy [22], Radiological joint assessment for early structural changes in joints are assessed using ultrasound [23] or magnetic resonance imaging (MRI) when feasible [24] as well as late osteochondral changes are assessed on plain radiographs (Pettersson score) [25].

**Table:** 

Recommendations (Panellist consensus)
- It is recommended that the frequency of all bleeds is documented in real-time by patients/caregivers through diaries or web applications when available, and reviewed together by the hemophilia treatment centre at least annually to calculate ABR, with reference to intra-articular, intramuscular and central nervous system bleeds, including their recovery status and the response to factor replacement -Any trained therapist or clinician can administer the FISH. It is advised that one reads the complete instructions before scoring the activity and that one performs the test as instructed, without any modifications. It is also necessary to score the activity only after observing the subject performing the task. One should not score the item based on the person’s subjective ability. Training and standardization of the technique is needed -Assessment and documentation of the musculoskeletal using HJHS and overall health of each patient should be done at least annually in adults and every 6 months in children

### Monitoring and management of pain

Patients with hemophilic arthropathy can suffer long-term adverse effects due to chronic pain, disability and reduced quality of life [26]. Monitoring for pain and its causes are essential to guide proper management [2, 27]. Pain assessment can be done using the qualitative& quantitative pain scales [28, 29]. For pain management, avoid non-steroidal anti-inflammatory drugs (NSAIDs), which are the mainstay of pain therapy in PWH; however, COX-2 inhibitors have been found to be relatively safe. COX-2 selective NSAIDs is contraindicated in people with established cardiovascular disease, cerebrovascular disease, peripheral arterial disease and mild-to-severe heart failure [26]. Paracetamol is the medicine of choice but does not have a very strong analgesic effect however, it is more effective if used regularly rather than as required, or when combined with an opioid such as codeine or dextropropoxyphene. In acute hemorrhage, ice cooling as part of the PRICE (protection, rest, ice, compression and elevation) protocol may alleviate pain. Using the principles of an analgesic ‘ladder’, common to pain relief guidance, medication should be prescribed with a stepwise progression from ‘simple’ paracetamol through to ‘strong’ opioid analgesics [30].

**Table:** 

Recommendations (Panellist consensus)
- Pain is best assessed and addressed in the context of a comprehensive care setting. A team that includes the hematologist, pain specialist and physical therapist should be involved in the pain assessment and management - Paracetamol is the medicine of choice but does not have a very strong analgesic and is more effective if used regularly rather than as required, or when combined with an opioid such as codeine or dextropropoxyphene - Careful monitoring for opioid-related side effects and the risk of dependence - COX-2 inhibitors have been found to be relatively safe yet contraindicated in patients with cardiovascular and cerebrovascular diseases - In acute haemorrhage, PRICE (protection, rest for 24-48 h, ice, compression 20 min at a time for the first 48-72 h and elevation) technique is recommended to alleviate pain

## 4-CNS bleeding

Central nervous system (CNS) bleeding is the most common emergent bleeding event encountered among PWH [31, 32] Mortality from intra cranial hemorrhage(ICH)is around 20% [33], and is higher in younger children and in developing countries [34]. The prevalence of ICH in the pediatric (non-neonatal) hemophilia population is about 12%, with 50% of cases being clinically silent [35]. Trauma is the leading cause of this devastating complication in childhood and adolescence [33]. Predisposing risk factors for spontaneous ICH include severe disease, infections (hepatitis C and HIV), the presence of inhibitors, age < 5 or > 50 years, a previous ICH episode [36], thrombocytopenia, hypertension [33, 37] and the absence of prophylaxis [38]. Decompression surgery by laminectomy for spinal hematoma may be needed at any age to relieve spinal cord compression and to reduce the risk of paralysis, especially if pressure is not relieved with clotting factor concentrates (CFCs) during the first critical hours. However, early identification of spinal hematomas and administration of CFC replacement therapy to achieve normal physiologic levels may prevent the requirement for neurosurgery [39]. 

### ICH in neonates

ICH incidence in neonates with hemophilia (3.4–4.0%) is substantially higher (40–80 times) than in neonates without congenital bleeding disorders. Nearly half of these ICH episodes occur in the first days of life and are frequently related to the delivery [40].

Suspicion of ICH calls for an immediate cranial ultrasound [31] However, cranial ultrasound is limited in its capacity to detect subdural or subarachnoid hemorrhages, especially at the convexity of the brain; hence, a computed tomography (CT) scan is preferred after traumatic delivery [41]. it has been recommended that all male newborns presenting with ICH should have an APTT test performed immediately after birth and that FVIII and FIX assays should also be conducted as soon as possible [42]. 

### Management considerations

For any head trauma or suspected CNS bleeding in PWH, if the patient is on home therapy, the product may be administered before leaving home or on route to the facility (preferably a hemophilia treatment center), provided the bleeding is not life-threatening and this does not result in delay [31]. The maximum time between arrival at the hospital and clinical assessment should not exceed 15 min, and the maximum time to clotting factor replacement should not exceed 30 min [43].

Sufficient CFC should be infused to ensure a normal physiologic level initially (at least 100% within the normal range). If neurosurgery is needed, subsequent doses should be given much sooner because of high factor clearance during surgery. Further doses will depend on imaging results; maintaining factor level until etiology is defined is essential [31]. Continuous infusion shortly after the bolus dose is appropriate. Alternatively, repeated doses can be given according to CFC availability [44]. Circulating factor levels should be always maintained above 50% for up to 3 weeks following a CNS bleed to minimize the risk of a re-bleed. A longer duration may be needed if an extensive neurosurgical procedure was required [4]. Hemophilia A patients who have experienced an intracranial hemorrhage (ICH) involves meticulous monitoring, factor replacement therapy, and potentially rehabilitation. A multidisciplinary approach, involving hematologists, neurologists, and potentially other specialists, is crucial for optimal outcomes [45].

**Table:** 

Recommendations(Panellist consensus)
-Any head trauma in PWH must be considered non-trivial until proven otherwise, and immediate treatment is recommended -Any unexplained or persistent headache, even in absence of history of head trauma, in PWH aged 3–18 years should be evaluated and managed -Delivery of a fetus with suspected hemophilia A should take place in a hemophilia A treatment center under the supervision of a hemophilia expert - A CT scan of the brain is the preferred method of evaluation -The factor level should initially be raised to 100% and maintained appropriately for at least 14 days. Regular monitoring of factor levels to ensure they are maintained within the target range - Lifelong or long-term secondary prophylaxis is mandatory after CNS bleeding. The decision should be based on the severity of the bleed, the risk of recurrence, and patient preferences - Suggested peak plasma factor levels and duration of administration in patients with CNS bleeding (with and without significant resource constraint) are adopted from WFH Guidelines (Table 3)

## 5-Hemophilia A carrier care

A carrier state should be suspected in a girl with abnormal bleeding and a positive family history of hemophilia, for example the daughter of a PWH should be considered an obligate carrier. = Around 30% of hemophilia A carriers display mildly reduced levels of 0.41 and 0.60 IU/mL that have been associated with bleeding [46–48].

Heterogeneity of symptoms in female carriers could occur due several variables that lead to low factor levels, including inactivation of one of the X chromosomes (known as Lyonization); ABO blood group; mutations in the genes encoding von Willebrand factor; compound heterozygosity, or homozygosity [46]. Carriers with FVIII/FIX levels in the normal range may never require factor replacement therapy. However, some carriers with factor levels in the lower range of normal (i.e., below 50 IU/dL) experience bleeding problems like males with mild hemophilia (e.g., hemorrhaging after dental extraction, surgery, or trauma) as well as problems that are specific to women, such as prolonged or heavy menstrual bleeding [4].

Bleeding in female carriers is mostly mucocutaneous bleeding, menorrhagia, bleeding with interventional procedures [46, 47]. It has been shown that between 14 and 19% of hemophilia A carriers report hemarthrosis with some showing pathologic and radiologic evidence of structural joint damage. Furthermore, studies have shown an association between FVIII or FIX deficiencies among hemophilia carriers and reduced range of motion in joints [49, 50].

**Table:** 

Recommendations(Panellist consensus)
-Immediate female relatives (mother, sisters and daughters) of a person with hemophilia A should have their clotting factor level checked whenever appropriate particularly prior to major procedures, surgery, or pregnancy - Carriers should be treated with a multimodal approach that enhances patient education and awareness using accessible brochures, pamphlets, or fact sheets that provide clear and concise information about menstrual health,, with an emphasis on self-report of symptoms and communication with healthcare providers - The need of Birth control pills and antifibrinolytic agents in controlling symptoms of menorrhagia among Hemophilia carrier Female.Some carriers with factor levels in the lower range of normal (i.e., below 50 IU/dL) who experience severe bleeding problems may require factor VIII replacement

## 6- acquired hemophilia

**Fig. 1:**
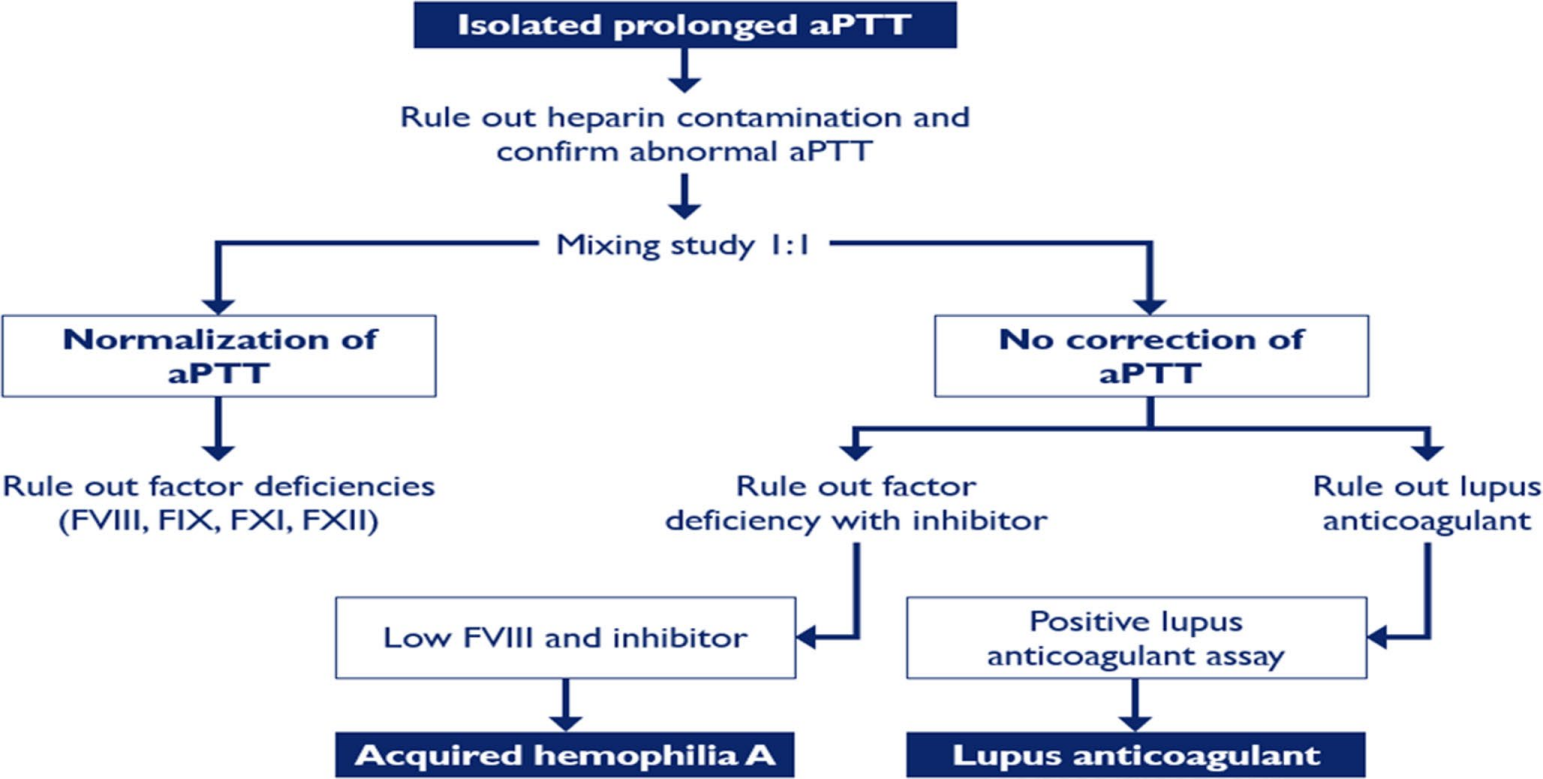
Diagnostic algorithm for acquired hemophilia (Tiede A el, Haematologica. 2020)

**Fig. 2:**
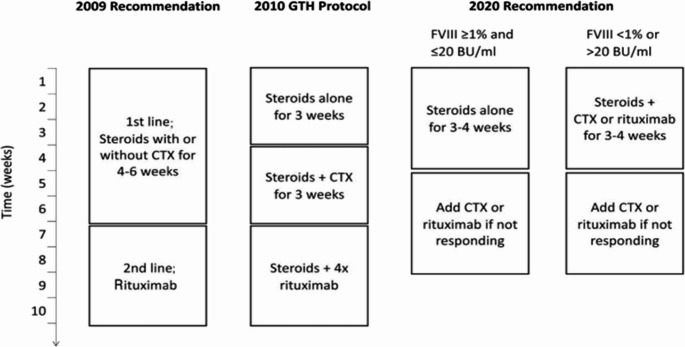
Reccomendations regarding immunosuppresive therapy in patients with acquired hemophilia A. Comparison of immunosuppresive therapy regimens recommended in the 2009 international recommendattions by Huth-Kulne et al.^1^ the GTH study^10^ and the current paper. FVIII; factor VIII activity; BU; Bethseda unit; CTX, cyclophosphamide (Tiede A et al, Haematologica. 2020)

Acquired hemophilia is a rare autoimmune disorder, which is caused by the production of inhibiting antibodies against a coagulation factor, most commonly factor VIII. It can occur in both males and females of all ages who have no previous history of abnormal bleeding events. It is also often associated with other underlying autoimmune disorders (e.g. rheumatoid arthritis, lupus), pregnancy and dermatological disorders. The severity of bleeding can vary ranging from no or mild bleeding to life-threatening bleeding events but unlike congenital hemophilia, hemarthrosis is rare [51, 52]. An algorithm for the diagnosis and managemnent of acquired hemophilia is shown in Figs. 1 and 2.

**Table:** 

Recommendations(Panellist consensus)
-Diagnosis is based on Correction and mixing studies using pooled normal plasma The APTT/Normal Plasma mix may be initially normal then progressively prolonged on incubation in time dependent antibodies -Bleeding patients with acquired hemophilia are treated using bypassing agents, including recombinant factor VII, activated prothrombin complex concentrate, or recombinant porcine FVIIII with the removal of autoantibodies by immunosuppressive therapy (eg. corticosteroids, rituximab, cyclophosphamide or a combinations of these agents)

## 7- Non factor replacement

New and emerging innovative therapeutics have been developed with alternative modes of delivery (e.g., subcutaneous), that overcome the limitations of current clotting factor replacement therapy (i.e., intravenous administration, short half-life, risk of inhibitor formation), and markedly increase compliance. Emicizumab mimics the cofactor activity of FVIII. It is administered subcutaneously once weekly, and in some cases can be administered more infrequently [53, 54]. The first, and the only licensed non-factor replacement therapy for hemophilia A in Egypt is Emicizumab.

### Indications

Several phase 3 clinical trials and post-marketing experience have shown that Emicizumab is effective prophylaxis in adults and children with inhibitors. Emicizumab reduces morbidity, complications, and hospitalization, and is cost-effective [54]. Inhibitor eradication by immune tolerance induction(ITI) therapy is successful in 70%−80% of patients with severe hemophilia A. Yet it requires frequent infusions, and a good venous access using a central venous access device (CVAD) which may be is associated with infection and/or thrombosis. For this reason, Emicizumab, which is administered subcutaneously and requires no IV access, has been considered a simpler option [55, 56]. 

WFH recommendations for patients with moderate/severe hemophilia A or B, who have experienced a life-threatening bleed (e.g., intracranial hemorrhage [ICH]), to start prophylaxis with FVIII or FIX concentrates or with a non-factor therapy (particularly important during the first 3–6 months following an ICH as the risk of recurrence is highest during this period as well as the risk for inhibitor development. Another WFH recommendation would be the use of Emicizumab for patients with hemophilia and venous access difficulties that impede regular clotting factor concentrate infusions factor making it easier to start patients on prophylaxis at an earlier age [4, 57].

**Table:** 

Recommendations(Panellist consensus)
- The use of non-factor therapy Emicizumab for inhibitor positive moderate/severe hemophilia patients - The panel recommend the use of non-factor therapy Emicizumab for patients with moderate/severe hemophilia who have experienced a life-threatening bleed (e.g., intracranial hemorrhage [ICH]) during the first 3–6 months following an ICH - The use of non-factor therapy Emicizumab for patients with hemophilia and difficult venous access - Prophylaxis dosing with Emicizumab consists of an induction period of 3.0 mg/kg/week for 4 weeks by subcutaneous injection. This is followed thereafter by 1.5 mg/kg/week or alternative dosing schedules including 3 mg/kg every 2 weeks or 6 mg/kg every 4 weeks -For acute bleeds in patients with hemophilia A and inhibitors on Emicizumab, It is recommended to use rFVIIa over aPCC.

### Monitoring while on Non-factor replacement

In patients receiving Emicizumab who have risk factors for thrombosis, e.g., past venous thromboembolism, obesity, smoking, chronic infection, or inflammation, rFVIIa should be used with caution due to the potential risk of acute non-STEMI and pulmonary embolism [58]. As Emicizumab interferes with the measurement of FVIII: C and FVIII inhibitors using the one-stage FVIII assay, a specific chromogenic assay using bovine reagents is used to detect inhibitors to FVIII [59, 60].

**Table:** 

Recommendations (Panellist consensus)
-The panelist recommends bovine chromogenic assays (bovine FX in kit reagent) to monitor inhibitor level for patients with hemophilia A and inhibitors who receive Emicizumab -The panelist recommends close clinical monitoring for thrombosis particularly thrombotic microangiopathy, adverse reactions, and thrombotic microangiopathy for patients with hemophilia A and inhibitors who receive Emicizumab

## 8- ESH achievement 2024

### Transitional and adult hemophila care

Challenges during transition to adulthood medical care include severing the relationships with the long-term pediatric care provider, adaptation to an unfamiliar environment, new responsibilities of self-management, miscommunications between the existing pediatric care provider and the new environment of care with the adult hemophilia care team, and barriers to availability of replacement factors [61].

Developing countries, such as Egypt, are faced with numerous challenges and barriers for the provision of care for adult patients with hemophilia and other RBDs, like the lower visibility of hemophilia patients in the healthcare system, the non-availability of factor concentrate and the lack of financial support to implement immune tolerance induction for inhibitor positive patients, the inadequate pain management, the lack of research and publications, challenges related to home care and home infusion, psychological barriers that can affect the adherence to treatment regimens [1]. 

ESH aimed to resolve the lack of care system for adults’ patients with hemophilia by coordinating with different specialties and subspecialities to assemble a multidisciplinary team.

ESH initiatives included the provision of medical care for less cost by universities, the development of medical insurance sponsored by the Ministry of Health (MOH) and the call for fund raising through donations from individuals and businessmen in the community, as well as non-governmental organizations (NGOs).

ESH developed training programs for the adult patients and their parents and caregivers about self-care, how to access trained caregivers who can handle emergency situations of mild to moderate musculoskeletal disorder. Nevertheless, raising awareness of physicians in the front-line of care will save the patient’s limbs and life through basic care of trauma and appropriate referral to any of the centers capable of managing hemophilia and RBDs.

### Patient advocacy in hemophilia

Health care advocates work closely with patients, their loved ones and other professionals working in the facility, focusing on health conditions, healthcare resources, and the needs of patients and the public. The presence of the Egyptian Society of Hemophilia (ESH) and its linkages with World Federation of Hemophilia was an important step in establishment of a strong advocacy program targeting government, doctors, and opinion makers to solve many of the challenges. This program provided hands-on training, education and awareness for all physicians involved in hemophilia care in all subspecialities, coaching, and tools to encourage as well as empower national member organizations (NMOs) to implement successful advocacy projects and a fair amount of personal contact with the key health and government officials who make the policy allocation decisions on health budgets.

## ESH strategic plans 2025

Future goals of ESH includes expanding membership to include people with Von Willebrand Disease and rare bleeding disorders, carriers of hemophilia, women with bleeding disorders in the registry and establishing guidelines for management and care for those patients. By including these other groups, we strengthen the society, we have more lobbying power, and we can call for greater resources to be spent in this area. Other plans include increasing the availability of FVIII from 0.36 IU/capita to 0.86 IU/capita, introducing a national screening program for inhibitors to FVIII and FIX, and increasing the governmental support for rehabilitation of patients with IBD.

## Data Availability

No datasets were generated or analysed during the current study.
